# The elastic modulus for maize stems

**DOI:** 10.1186/s13007-018-0279-6

**Published:** 2018-02-08

**Authors:** Loay Al-Zube, Wenhuan Sun, Daniel Robertson, Douglas Cook

**Affiliations:** 1 Division of Engineering, New York University-Abu Dhabi, P.O. Box 129188, Abu Dhabi, United Arab Emirates; 2 Faculty of Engineering, The Hashemite University, P.O. Box 330127, Zarqa, Jordan; 3 Department of Mechanical Engineering, University of Idaho, 875 Perimeter Drive, MS 0902, Moscow, ID 83844-0902 USA; 4 Department of Mechanical Engineering, Brigham Young University, 435 Crabtree Building, Provo, UT 84602 USA

**Keywords:** Stalk lodging, Modulus, Bending, Compression, Tension, Maize

## Abstract

**Background:**

Stalk lodging is a serious challenge in the production of maize and sorghum. A comprehensive understanding of lodging will likely require accurate characterizations of the mechanical properties of such plants. One of the most important mechanical properties for structural analysis of bending is the modulus of elasticity. The purpose of this study was to measure the modulus of elasticity of dry, mature maize rind tissues using three different loading modes (*bending*, *compression* and *tensile*), and to determine the accuracy and reliability of each test method.

**Results:**

The three testing modes produced comparable elastic modulus values. For the sample in this study, modulus values ranged between 6 and 16 GPa. All three testing modes exhibited relatively favorable repeatability (i.e. test-to-test variation of < 5%). Modulus values of internodal specimens were significantly higher than specimens consisting of both nodal and internodal tissues, indicating spatial variation in the modulus of elasticity between the nodal and internodal regions.

**Conclusions:**

Bending tests were found to be the least labor intensive method and also demonstrated the best test-to-test repeatability. This test provides a single aggregate stiffness value for an entire stalk. Compression tests were able to determine more localized (i.e., spatially dependent) modulus of elasticity values, but required additional sample preparation and test time. Finally, tensile tests provided the most focused measurements of the modulus of elasticity, but required the longest sample preparation time.

## Background

Maize is one of the world’s top crops and is used in hundreds of applications worldwide. But annual losses due to late season stalk lodging of maize is a serious challenge that hinders economic effectiveness of maize production. In particular, the problem of late-season stalk lodging (breakage of the stalk prior to harvest but after reproductive stage six) has been estimated to reduce worldwide corn yields by 5–20% [1, 2]. The complete characterization of structural and material properties of maize stems should enable a more comprehensive understanding of stalk lodging [3]. However, a prerequisite to this approach is to first develop and validate accurate methods of measuring the mechanical tissue properties of maize stalks. Of the many tissue properties that can be measured, the longitudinal modulus of elasticity is perhaps one of the most important for structural bending analyses [4].

Determining the elastic modulus of the rind tissue of maize stems is challenging for several reasons. First, the irregular shape of maize stalks makes the specimens difficult to handle with standard mechanical test equipment. Second, the dependency of elastic properties of plant tissues on their state of maturation and moisture content has a strong effect on measured properties [5]. Systemic testing, thus, would require control of maturity and moisture content during, preparation, storage, and testing. Third, the variability of biologic material requires testing of several specimens in order to statistically characterize untested parameters dictated by nature and not controlled in engineering environments. Thus, results from mechanical testing of biologic materials have an element of error that is not often quantified. Finally, maize stems consist of a complex structure whose various components are intimately connected. Thus, often only bulk material properties can be easily measured.

**Fig. 1:**
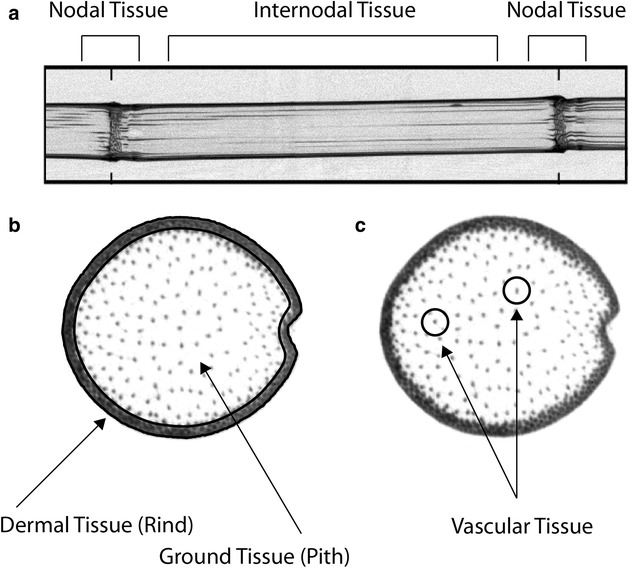
The anatomy of corn stalk. **a** Lateral view of a maize stalk with nodal and internodal tissue regions identified. **b** Transverse cross-section of a maize stalk with dermal (rind) and ground (pith) tissues identified. **c** Transverse cross-section of a maize stalk with the vascular tissue identified. Images obtained by X-ray computed tomography

Maize stems are composed of three primary tissue types shown in Fig. 1: (1) dermal tissue or “rind” as a strong protective surface layer; (2) ground tissue (pith), which is softer and makes up the largest fraction of a stem’s volume; and (3) vascular tissue that adds structural support and provides water and nutrient transport [6–8]. The rind is composed collenchymas, parenchyma, and sclerenchyma with the collenchymatous and sclerenchymatous tissues providing the principle structure supporting cells against tension and bending loads. The pithy core of the stem is made of a compliant material composed mainly of parenchyma. The pith acts much like foam cores in sandwich-structured composites in that it provides resistance to buckling of the stem [7, 9]. Periodic nodes located along the length of the stalk likewise act to restrain the stem against compressive buckling [10] and provide transverse reinforcement. A more detailed description of the anatomy of plants stems is found in Shah et al. [5].

Due to the complex structure of maize stalks as well as the above-mentioned testing challenges, biomechanical data on the material properties of maize stalk tissues are currently limited to a handful of studies. These have included the determination of the compressive modulus of elasticity using dry internodal specimens with a length-to-diameter ration of 1:1 [11], bending modulus of elasticity of moist maize internodal specimens sampled at the pre-tasselling and dough growth stages [12, 13], the tensile modulus of elasticity of moist and dry rind tissue of maize stalks [14], and a reduced modulus of elasticity (indentation modulus) of dry rind fibers using nanoindentation [15, 16]. Most of these studies utilized only a single test method, and the reported values for rind modulus varied between 0.26 and 20 Gpa.

The goal of the present study was to obtain modulus of elasticity values of maize rind tissue by using a variety of mechanical loading modes to enable comparisons between these methods. Since maize is susceptible to late-season stalk lodging, dry, mature maize stalks were utilized in this study. Three loading modes were evaluated: transverse 3-point bending, longitudinal compression, and longitudinal tensile loading. From these tests, a total of 5 elastic modulus values were obtained. Direct comparisons between the values obtained by each method were used to determine the accuracy and reliability of each method, as well as the advantages and disadvantages of each testing method.

## Methods

### Experimental design

**Fig. 2:**
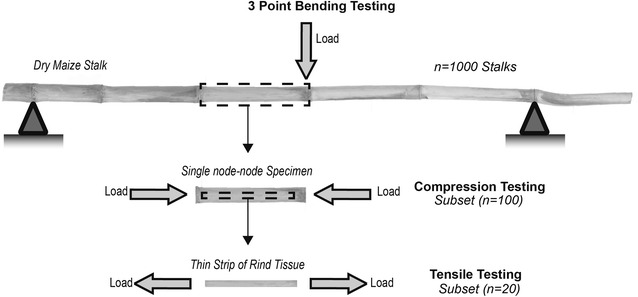
A diagram depicting test specimens structures used in each loading mode. Stalks used in 3-point bending testing (top), internode specimens used in compression testing (middle). Rind tissue specimens used in tensile testing (bottom)

A paired-comparison experimental design was used in this study to reduce the effects of inter-specimen variability. Specimens were tested in three modes: bending, compression and tension. As illustrated in Fig. 2, bending tests were first performed on stalk sections consisting of several internodes. Next, single internode sections were cut from each stalk for compression testing. Finally, thin strips were excised from the compression test samples for tensile testing.

Sample sizes for each test type varied in proportion to the time required for test preparation and testing. A set of 1000 stalks was subjected to bending tests, a subset of 100 stalks were then tested in compression, and 20 thin tissue strips from 10 of the compression samples were selected for tensile testing. The following sections describe the stalk samples, specimen preparation, testing equipment, and the testing procedures that were used in this study.

### Quantifying geometric properties of stalks

X-ray computed tomography (CT) was used to quantify geometrical properties. Stalks were scanned using an X5000 scanner (NorthStar Imaging, Rogers, MN, USA). The scanning process produced 2D cross-sectional images of the maize stalks. A customized computer program was used to extract cross-sectional areas of the rind and pith regions as well as the area moment of inertia from the CT data. The Cross-sectional area and the area moment of inertia were obtained in the constant geometry region of the internode directly above the loaded node in the three-point bending test. The scanning and morphology extraction are described in more detail in previous studies [17, 18]. It should be noted that using only a single value for the area or the moment of inertia of the stalk introduces some error in the estimation of the elastic modulus (i.e., stalks are tapered structures and both the diameter and moment of inertia changes along their length). However, for bending tests, this error has been shown to be negligible [19].

### Plant material

Dry, mature maize stalks (10–15% moisture by weight) were used in this study to mimic the state of stalks in the field just prior to harvesting (Table 1). It is at stage of development (dry and mature) that stalks are most susceptible to late-season stalk lodging. Stalks were sampled from 2 replicates of five commercially available hybrids of dent corn (maize) seeded at 5 planting densities (119,000, 104,000, 89,000, 74,000, and 59,000 plants ha^−1^). Additional information about the origin and sampling of these stalks can be found in a previous report [17].

**Table 1:** General conditions: length and aspect ratio of test specimens

Group	Length (mm)	Aspect ratio (length/width)
Bending (n = 1000)	950.84 ± 150.55	46.36 ± 9.26^a^
Compression (n = 100)	186.82 ± 26.11	9.64 ± 1.59^a^
Tensile (n = 20)	80.26 ± 1.67	25.81 ± 4.34^b^

The data represents average values ± standard deviation

^a^Aspect ratio with respect to the major diameter obtained via CT scanning

^b^Aspect ratio with respect to the specimen width measured using caliper

### Mechanical testing equipment

All tests were performed in a laboratory setting using an Instron universal testing machine (Model 5965, Instron Corp., Norwood, MA). Instrumentation control and data acquisition were managed with Instron software (Bluehill 3.0). All tests were displacement controlled, with force and displacement measured synchronously during the test. Low levels of deformation were applied in each test to avoid physical damage to test specimens. Maximum deformation and strain values of each test type are given below.

### 3-Point bending test

As shown in Fig. 2, maize stalks were supported at the initial and terminal nodes of each stalk, with a load applied at the node closest to the center of the stalk. It should be noted that this approach often dictates a non-symmetric loading scenario which is properly accounted for in Eq. 1. Stalks consisted of between 4 and 7 internode segments. A 500-N load cell was used to collect the force data at a frequency of 10 Hz, and stalks were deflected < 6 mm at a constant rate of 1.666 mm/s. This procedure ensured the test didn’t induce any permanent damage to the stalk. Additional details about the bending test methodology can be found in previous reports [20, 21].

Bending elastic moduli ($E_{bending}$) of maize stems were calculated utilizing the principles and approximations of engineering beam bending theory [22]. The slopes of bending force–deflection ($\Phi$) curves and the area moment of inertia (I) of maize stems were determined as described previously [17, 18, 21], and were employed in calculating $E_{bending}$ using Eq. 1 (see “Appendix 1”).

1 $$ E_{bending} = \frac{{a^{2} b^{2} }}{3IL} \varPhi $$

In this equation, (*L*) is the distance between the left and right supporting anvils, (*a*) and (*b*) are the distances from the left and right anvils to the point of the applied load, respectively, and (*I*) is the area moment of inertia of the rind tissue. The structural contribution of the pith tissue is ignored in this approach. Previous reports indicate that this assumption does not introduce significant errors [10, 17, 20, 21], and it should be noted that the calculation of the rind modulus cannot be accomplished from a single test without this assumption.

### Compression tests

Longitudinal compression tests were performed on a subset of 100 internodal specimens (Fig. 2). The internodal specimens used were prepared from stalks picked randomly from the 1000 stalk set used for bending testing. The internodal specimens were cut just below and above each node [23]. Compression tests were conducted using two self-aligning platens (Cat No: S5722A, Instron Corp., Norwood, MA, USA), an extensometer with a reference length of 50 mm (Instron 2630 Series Dynamic Extensometer, Instron Corp., Norwood, MA, USA), and a 5-KN Instron load cell. The displacement rate was 0.1 mm/s and the sampling frequency was 33 Hz. Slope measurements were obtained by inducing < 0.35 mm of specimen deflection (strain values < 0.5%). This procedure ensured the tests did not induce any permanent damage to the specimens.

As previously reported [23] two estimates of elastic modulus can be obtained in a compression test of this nature; *overall* and *local*. *Overall* moduli were calculated using strains based on the displacement of the uppermost compression platen and the entire length of the specimen. *Local* moduli were calculated using the deformation recorded between the two arms of the extensometer. The *overall* compressive modulus represents a single aggregate value for all rind tissue in the internodal specimen and can be obtained relatively quickly since the extensometer is not required. The *local* compressive modulus provides a more focused measurement because it measures deformation within a limited region of the specimen. However, accurately measuring the local modulus generally requires conducting multiple axial tests. Further details on the *local* and *overall* modulus methodology are available in a previous paper [23].

### Tensile tests

Technical standards govern the processes for evaluating mechanical parameters of various synthetic materials as well as lumber. But few such standards exist for testing biological tissues such as corn stalks. Nevertheless, existing standards can provide useful guidelines for the measurement of such tissues. For example, ASTM D4761-13 recommends that tensile specimens be constructed with an aspect ratio (length/width) > 12 [24]. This choice of aspect ratio helps minimize in influence of end effects. The aspect ratio of tensile specimens in this study was between 18 and 20.

Longitudinal tensile tests were performed on thin strips of rind tissue that were carefully dissected from compression-test specimens. The compression test specimens were first cut transversely with a rotary electric saw. Next, thin strips were cut lengthwise with a razor. The inner face and lateral sides of these thin strips were gently abraded to remove pith tissue and to improve geometric homogeneity of test strips. The length of prepared specimens ranged from 151.5 to 157 mm. The width of specimens ranged from 2.13 to 4.25 mm.

**Fig. 3:**
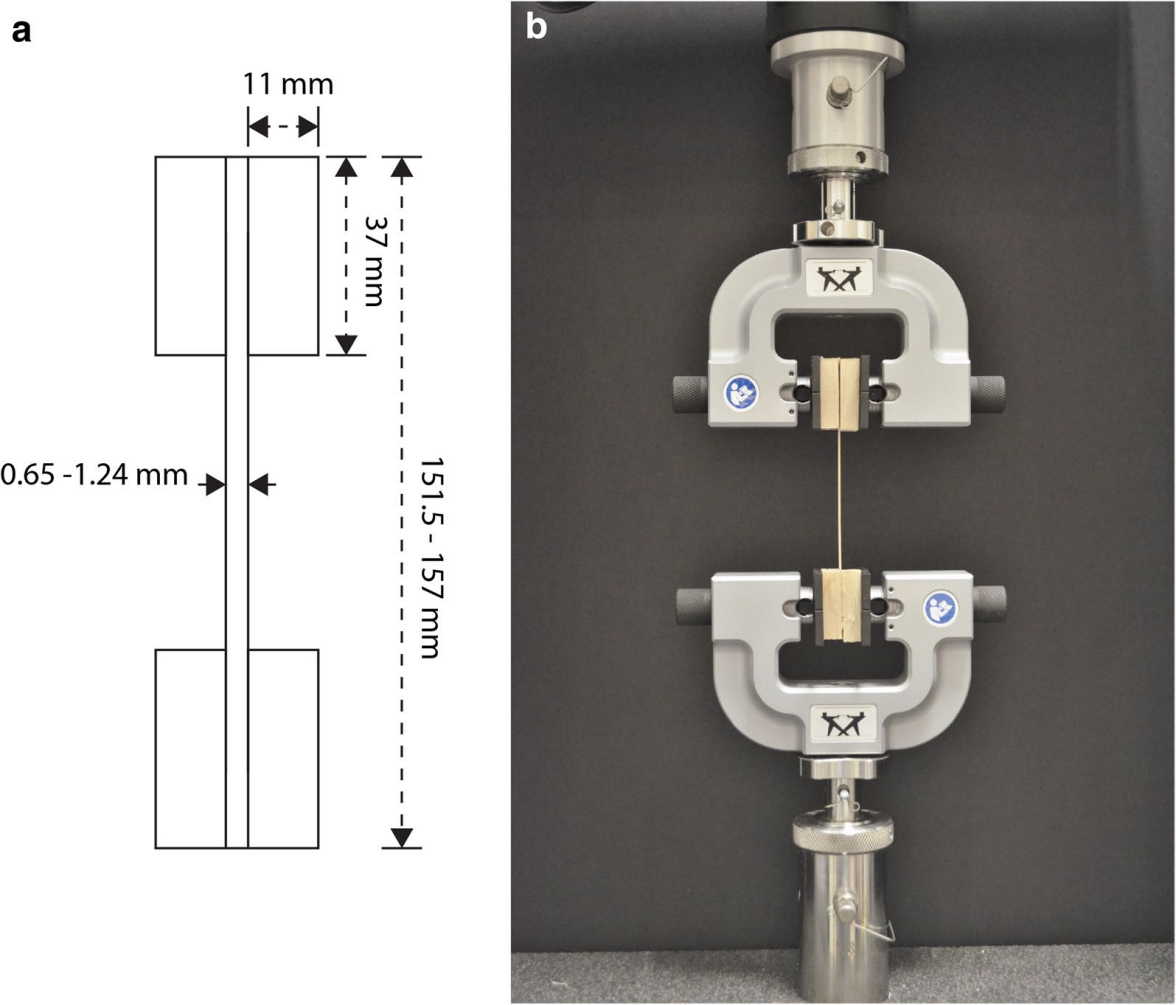
Tensile testing setup: **a** schematic diagram depicting geometric features of an ideal thin strip specimen of rind tissue; **b** a photograph of one specimen situated for testing

Secure gripping is a common challenge when testing biological tissues because the gripping technique must prevent slippage without inducing damage to the specimen. This was accomplished in this study through the use of adhesive and wooden blocks. Both sides of rind strips were glued to plane square edge (PSE) rectangular blocks of softwood (approximately 37 mm by 21 mm by 11 mm). A commercial cyanoacrylate glue was found to produce the most secure adhesion (Bison International, The Netherlands). After mounting, the specimen length between wood blocks ranged between 77.5 and 83 mm (Fig. 3a).

After the adhesive was fully cured, test specimens were mounted in the test fixture using side action fixed grips (Cat No: 2710-114, Instron Corp., Norwood, MA, USA). Serrated grip faces provided secure connection between the grips and the wood blocks. A specimen mounted for testing is shown in Fig. 3b.

A 5-KN load cell (Cat No: 2580-108, Instron Corp., Norwood, MA, USA) was used to collect the force data at a frequency of 33 Hz, and a deformation rate of 0.1 mm/s was used. The loads used in this test typically resulted in strain values < 0.5%. The displacement rate used is within range recommended for testing the tensile axial strength of lumber and wood-base structural materials [24].

Strain was calculated based on the distance between wood blocks and the displacement of the jaw fixtures. This approach was chosen to eliminate non-axial deformation which was induced when an extensometer was attached to the thin specimen. The validity of this approach was also confirmed experimentally: extensometer measurements were found to be virtually identical to those obtained from the displacement of the jaws.

When testing biological tissues, a preload and repeated application of load cycles is commonly used to bring the samples to a repeatable reference state [25]. Therefore data was collected after applying an initial load of 10 N to each specimen. Five loading cycles were then applied, where in each cycle the load increased from 10 to 110 N and then returned to the 10 N initial state. Only measurements from the latter four loading cycles were employed in the tensile modulus calculation. This procedure did not induce any damage to the specimen. More information about the preloading and loading procedures can be found in [23].

Tensile elastic modulus is defined as the slopes of the corresponding stress–strain curve. The tensile stress, σ, was obtained by dividing the applied force (*F)* by the cross-sectional area of the rind strip (*A*_*r*_*)*. For each rind strip specimen, the cross-sectional area employed in the stress evaluation was estimated twice; using X-ray computed tomography and using a caliper. X-ray computed tomography areas were calculated using a customized computer program to estimate the cross sectional area of 2D X-ray images from the middle portion of test specimens. Cross-sectional areas obtained via calipers were accomplished by measuring the cross-sectional area of specimens at three distinct locations (i.e., from the top, middle, and bottom portions of each specimen). These three measurements were then averaged to obtain the cross-sectional area of the specimen. For small deformation such as those present in this study, strain can be obtained by dividing the change in length by the original length of the rind strip specimen prior to the 10 N pre-loading. Depending on the way cross-sectional areas were estimated, 2 tensile elastic moduli values were calculated and reported; E_tensile_ and E_tensile-caliper_. The former obtained using X-ray images and the later using caliper measurements.

### Repeatability and statistical analysis

When testing biologic materials, geometric and structural variations introduce an element of error that increases results uncertainty. Repeatability is one way to quantify uncertainty. Therefore, the repeatability of each test methodology used in this study was assessed. The design of this experiment consisted of 3 test types (bending, compression, and tension), 10 specimens per test type, and 5 repeated measurements per specimen. This structure resulted in 50 individual measurements for each method of determining the elastic modulus (bending, compression, and tension). The standard deviation was used to quantify test-to-test repeatability for each specimen following standard procedures [26].

Statistical analyses included unpaired and paired t tests. Unpaired tests were used to identify differences between testing methodologies, while paired tests were used to identify differences among the elastic moduli values obtained. For bending, compression, and tensile tests, the corresponding sample sizes were 1000, 100, and 20, respectively. Paired data (i.e., tests performed on specimens from the same stalk) was obtained for 10 stalks. Significance was established at *p* ≤ 0.05.

## Results

### Repeatability analysis

Recall that standard deviation was used to quantify test repeatability (i.e., test-to-test variation of a single specimen). With 10 samples tested 5 times each, all tests were found to have mean repeatabilities of less than (5%), with the majority of repeatability values under 10%. Repeatability data for each method is summarized in Table 2.

**Table 2:** Repeatability statistics obtained from repeated tests of prepared specimens: 10 stalk specimens, 10 internodal specimens, and 10 tensile specimens

Test type	Specimens description	Tissues tested	% Repeatability (Mean ± SD)	95% Successive tests confidence interval upper bound (%)	n
Bending	Stalks	Multiple	1.5 ± 0.9	3.7	10
Compression (*overall*)	Node–node	Multiple	3.8 ± 3.7	11.0	9^a^
Compression (*local*)	Node–node	Single	3.9 ± 2.7	9.2	9^a^
Tensile	Rind strips	Single	1.9 ± 1.3	4.9	10

Five tests were performed on each specimen to obtain a single reliability value, defined as the standard deviation of the resulting modulus of elasticity values

^a^One sample was damaged during testing and therefore was excluded

### Linear stress–strain curves

**Fig. 4:**
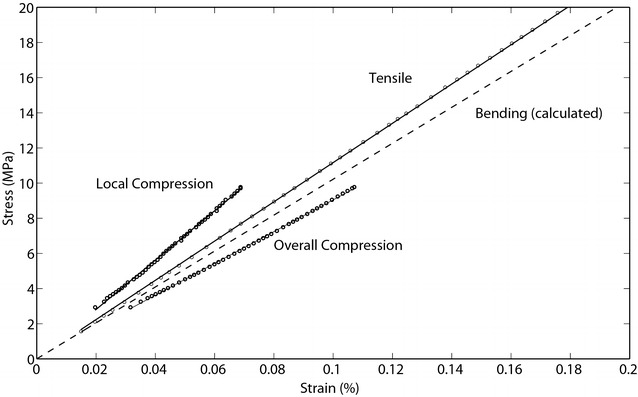
Bending, compressive and tensile stress–strain curves obtained from specimens originating from an individual maize stalk. Slopes of each curve represent the respective value E_*Bending*_, E_*Compression*-*local*_, E_*Compression*-*overall*_, and E_*Tensile*-*caliper*_

To prevent specimen damage, tests in the study were performed with strain values < 0.5%. Each test type produced stress–strain curves which were highly linear (*R*^2^ typically above 0.99). For a specific material, the elastic modulus is the slope of the stress–strain curve within small deformations (strains). Stress–strain curves of specimens originating from a typical maize stalk are shown in Fig. 4.

### Bending versus compression versus tensile elastic moduli comparison

**Fig. 5:**
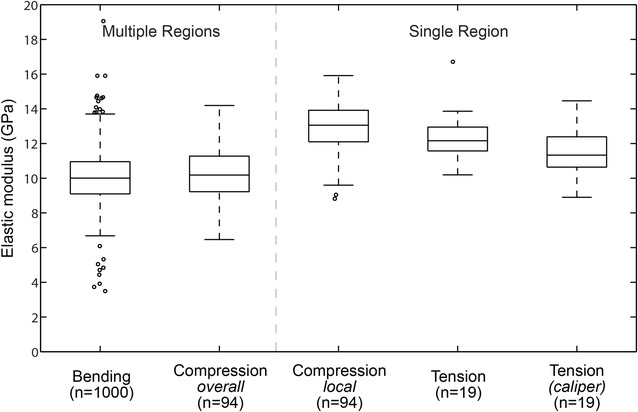
Elastic moduli distribution plots of all tests performed. Whiskers indicate the minimum and maximum moduli values obtained from each test, which are not considered outliers. Outliers are plotted individually using (°) symbol (MATLAB R2014a, MathWorks, Natick, Massachusetts, USA)

The vast majority of elastic modulus values ranged between 6 and 16 GPa. Distribution plots for all tests performed in this study are shown in Fig. 5. Recall that we conducted 3 different tests (bending, compression, tension) that produced 5 modulus values, 2 of which are obtained from compression testing and 2 from tensile testing. The mean and standard deviation values for *E*_*Bending*_, *E*_*Compression*-*overall*_, *E*_*Compression*-*local*_, *E*_*Tensile*_ and *E*_*Tensile*-*caliper*_ were 10.06 ± 1.51 GPa, 10.15 ± 1.47 GPa, 12.87 ± 1.56 GPa, 12.35 ± 1.51 GPa and 11.54 ± 1.37 GPa, respectively. One-way ANOVA analysis of the elastic moduli among all groups indicated that the methods are statistically different. These differences are more clearly understood when we look at paired data (see below).

**Fig. 6:**
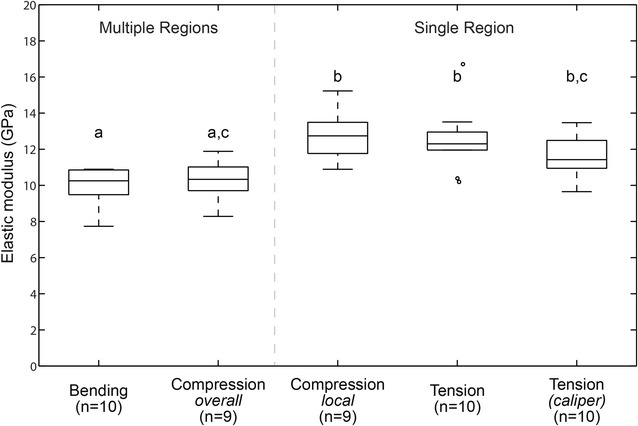
Elastic modulus distribution plots of the elastic moduli for paired specimens obtained from 10 maize stalks. No significant differences were observed in modulus values between bending and compression-*overall* groups (**a** *p* = 0.58), or between compression-*local* and tensile groups (**b** *p* = 0.4859), or between compression-*local* and tensile-*caliper* groups (**b** *p* = 0.051), or between compressive-*overall* and tensile groups (**c** *p* = 0.13) [Paired t tests]. However, bending modulus values were significantly lower than the compression-*local* and tensile values (*p* = 0.0001 and 0.038, respectively) [Paired t tests]. Data in the chart is from paired specimens obtained from 10 maize stems, 1 specimen was damaged during compression testing and therefor was excluded. Whiskers indicate the minimum and maximum moduli values obtained from each test, which are not considered outliers. Outliers are plotted individually using (°) symbol (MATLAB R2014a, MathWorks, Natick, Massachusetts, USA)

Two distinct groups for the modulus were identified when analyzing paired data; specimens involving nodal and internodal tissues (group I) and specimens involving only internodal tissue (group II). Distribution plots for paired specimens obtained from 10 maize stalks are shown in Fig. 6. Paired t tests did not reveal any statistical differences between bending and compressive-*overall* elastic moduli (a). Similarly, they did not reveal any statistical differences between compressive-*local* and tensile moduli values (b). Group I measurements (bending and compressive-overall values) represent a holistic estimate of the modulus of rind tissues of the entire stalk, while group II measurements (compressive-local and tensile values) represent an estimate of the modulus of rind tissues within a single internodal section of the stem. The paired mean and standard deviation values for *E*_*Bending*_, *E*_*Compression*-*overall*_, *E*_*Compression*-*local*_, *E*_*Tensile*_ and *E*_*Tensile*-*caliper*_ were 9.98 ± 1.02 GPa, 10.26 ± 1.06, 12.81 ± 1.3 GPa, 12.22 ± 1.31 and 11.54 ± 1.32 GPa, respectively.

## Discussion

### Accuracy

The accuracy of tests such as these is difficult to establish because the exact value of the modulus of elasticity of each sample is unknown. In addition, biological tissues can be notoriously difficult to measure accurately. For example, a previous study used bending, compression, and tension test methods to compare the modulus of elasticity of barley and wheat stems [11]. The study reported a modulus value of 7.3 GPa in tension and of 0.6 GPa in compression for the same wheat variety, values which correspond to percent discrepancies of (− 92%) and (+ 1117%), depending on which value is assumed to be correct.

However, all tests in this study produced modulus of elasticity values that were in the neighborhood of 12 GPa. For tests involving similar tissue types, the average discrepancy between median results was < 5% with a maximum discrepancy of 11%. The consistency of the tests in this study is one way to establish the accuracy of measured values.

Previous studies have reported different values for the modulus of elasticity of the rind tissue of dry maize. The lowest estimate in the literature was 0.26 GPa obtained using internodal specimen with a length-to-diameter ratio of 1:1 under compressive loads [11]. The maximum estimate was 20 GPa obtained using dogbone shaped specimens in tension [14]. In this study, straight-sided specimens were used for tensile testing as recommended when testing fiber reinforced materials having comparable dimensions [27].

### Modulus of elasticity and tissue regions

Specimens in this study can be divided into two groups. Specimens in the first group consisted of both nodal and internodal tissues. Both bending tests and overall compression tests utilized nodal + internodal specimens. The second group of specimens consisted of internodal tissues only. Local compression tests and tensile tests utilized these specimens. As shown in Fig. 6, the measured value of elastic modulus tended to be different between these two groups. Previous studies have noted spatial variation in material properties, both between nodes [21, 28], and basal/apical variation between internodes [29]. Because bending tests and overall compression tests utilize both nodal and internodal tissues, modulus of elasticity values obtained from these tests should be considered as aggregate values. On the other hand, local compression and tensile test methods utilize only internodal tissues, and therefore produce values that are likely more accurate than bending and overall compression tests. However, they are only valid for the tissue region from which they were taken.

The modulus of elasticity values of internode tissues were consistently higher than the values within the nodal + internodal tissues group. This suggests that internodal tissues have, on average, a higher modulus of elasticity than nodal tissues. This observation is consistent with the fact that internodal tissues are more organized and regular than nodal tissues [10, 21], and with previous work on nodal + internodal differences observed during bending tests [28].

### Symmetry in elastic moduli

Although the elastic modulus is typically assumed to be symmetric (i.e., the same modulus value in both tension and compression), asymmetric moduli of plant tissues have been reported [11, 30, 31]. The specimens in this study consistently exhibited symmetry of the modulus of elasticity. This was evidenced in two ways. First, for nodal + internodal specimens, the modulus values obtained from bending tests (which involved both tensile and compressive strain) were similar to those obtained in compression, which involved only compressive strain. Second, for tests involving only internodal tissues, tensile and compressive tests yielded similar results. Future modeling studies can therefore utilize an assumption of symmetric moduli. Furthermore, modulus of elasticity values from any of these tests can be used (as appropriate) in such modeling studies. The symmetry obtained in this study supports the convection that measurements errors may be the cause of the asymmetry of the elastic moduli values reported in the previously mentioned studies.

### Advantages and disadvantages of the various testing methods

The advantages and disadvantages of each testing method are discussed in this section. One common advantage is that all tests exhibited relatively favorable repeatability (i.e. test-to-test variation of < 5%). However, sample preparation and testing time varied widely between tests. Bending tests were the fastest and the most reliable tests; however this test provides only a single modulus of elasticity value. The obtained value is aggregated over the entire specimen and therefore obscures any spatial variation in stiffness. This aggregated-value is the one needed when modeling a real stem macroscopically. The overall compression test requires somewhat more sample preparation, but provides a modulus of elasticity that is aggregated over a single node-to-node specimen. If additional material specificity is required, the local compression test can be used. This approach provides the modulus of elasticity of internodal tissue, but requires repeated testing, as described in a previous study [23]. The total testing time of the local compression test is therefore higher than the overall compression test. Both compression test methods exhibited the worst test-to-test repeatability values. Finally, tensile tests are relatively fast and provide the most specific modulus of elasticity values, but require the longest specimen preparation time. The advantages and disadvantages of the four testing methods are summarized in Table 3.

**Table 3:** Comparison of the different testing modes used to obtain the elastic modulus: testing time estimates, geometry assessment tool, and the repeatability variation

	Preparation time min./specimen	Testing time min./specimen	Prep + Testing time min./specimen	Geometry assessment tool	% Repeatability mean (std)
Bending	~ 1 min	~ 2 min	~ 3 min	Micro-CT	1.5 (0.9)
Compression (overall)	~ 3 min	~ 3 min	~ 6 min	Micro-CT	3.8 (3.7)
Compression (local)		~ 10 min	~ 13	Micro-CT	3.9 (2.7)
Tensile	~ 40 min	~ 2 min	~ 42 min	Micro-CT, Caliper	1.9 (1.3)

The preceding discussion omitted the issue of geometric assessment. This issue is important because specimen geometry has a strong influence on the calculation of elastic modulus. In the current study, X-ray computed tomography (micro-CT) was used to assess test specimen geometry of most tests. The exception was the caliper-based tensile test (see Fig. 6). Micro-CT scanning requires expensive equipment and can be time consuming. On the other hand, it enables a highly detailed assessment of specimen geometry. The accurate assessment of specimen geometry is one reason that the repeatability values in this study were relatively low. The time associated with micro-CT scanning was excluded from Table 3 because it is highly dependent on the system being used. Alternatives to micro-CT scanning include measurement with hand tools such as digital calipers or obtaining cross-sectional images obtained with a flatbed scanner [32]. Flatbed scanning would provide more consistent measurements, but would require more sample preparation and computer aided digital analysis. On the other hand, digital caliper measurements can be collected quite quickly, but are be significantly less reliable.

### Limitations

The data in this study were obtained using dry, mature maize stalks and tests were focused on assessing the modulus of elasticity of the rind tissue only. As such, the modulus of elasticity values reported in this study are relevant only to similar tissue and specimens. For bending and compression tests, the modulus of elasticity was calculated using equations that effectively neglect the structural influence of the pith tissue. Previous research by the authors indicates that this assumption effectively *increases* the calculated modulus of elasticity by about 3–5% [23]. The data from those tests should therefore be viewed as lower bound estimates for the modulus of elasticity of dry, mature maize rind tissues.

Reliability estimates reported in this study involved repeated testing, but did not involve other sources of uncertainty. Other potential sources of uncertainty include sample preparation, the assessment of specimen geometry, the effect of shear, and the possible effect of indentation or section ovalization. Test-to-test variation due to these factors may be worth studying in the future. Deformation of the testing apparatus in this study was was negligible.

**Fig. 7:**
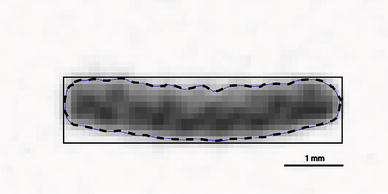
Micro-CT image illustrating differences between measuring the cross sectional area of tensile test specimens. The dashed line represents the specimen boundary identified through image processing while the solid lines represent the dimensions that would be expected by using digital calipers

As an example of uncertainty associated with sample preparation, consider Fig. 7, which contains a micro-CT cross-section of one tensile specimen. The dashed line represents the specimen boundary identified through image processing while the solid lines represent the dimensions that would be expected by using digital calipers. As shown in the figure, the cross-sectional area obtained by these methods differs by approximately 10%. In general, caliper measurements *overestimated* the cross-sectional area, which resulted in an *underestimation* of the modulus of elasticity values for the caliper method (see Fig. 6). Micro-CT images were used to assess this uncertainty because they are faster to obtain as compared to images of stained microscopic sections.

In addition to these factors, specimens typically exhibit slight variation in cross-sectional area in the axial direction. The reader should be aware that the reliability of any individual measurement is dependent upon all relevant sources of variation and uncertainty. In most cases, the overall reliability of these tests will be higher than the test reliability values reported in Table 2.

## Conclusions

This study compared the modulus of elasticity values of the rind tissue of dry maize stalks obtained using different mechanical loading modes (*bending, compression & tensile*), and contrasted the accuracy and reliability of these same test. All methods produced modulus of elasticity values that were within the range 6–16 GPa, indicating that each of these methods is effectively measuring the same physical feature of the maize rind, and that each method is relatively accurate. All methods exhibited good test-to-test repeatability values.

Bending tests were found to be the fastest and most repeatable measurement approach. However, because the bending test requires a long slender specimen, it produces an aggregate estimate of the modulus of elasticity. Compression tests require somewhat more sample preparation and testing time, but provide modulus of elasticity values that are more localized. Finally, the tensile testing approach is the most intensive in terms of sample preparation, but produces the most localized measurement of the modulus of elasticity. This information can be used in creating structural models of maize stalk lodging, and to guide future experiments.

## Appendix 1

According to engineering beam bending theory [22]:

$$ \begin{aligned} y & = \frac{{F a \left( {L - x} \right)}}{6 E I L} \left( {2 L x - x^{2} - a^{2} } \right) \\ at x & = a,   y = \frac{1}{3} \frac{F a \left( b \right)}{{E I \left( {a + b} \right)}} \left( {\left( {a + b} \right)a - a^{2} } \right)  \\ y & = \frac{1}{3} \frac{F a b }{E I L} \left( {a b} \right) \\ E I & = \frac{1}{3} \frac{F a b }{y L } \left( {a b} \right) \\ \end{aligned} $$

The slope for the force–deflection curve $\varPhi$ can be expressed as

$$ \varPhi = \frac{3EIL}{{a^{2} b^{2} }}\,thus\,E = \frac{{a^{2} b^{2} }}{3IL} \varPhi $$
